# Biocomposites Based on Polyamide 11/Diatoms with Different Sized Frustules

**DOI:** 10.3390/polym14153153

**Published:** 2022-08-02

**Authors:** Marta Dobrosielska, Renata Dobrucka, Paulina Kozera, Rafał Kozera, Marta Kołodziejczak, Ewa Gabriel, Julia Głowacka, Marek Jałbrzykowski, Krzysztof J. Kurzydłowski, Robert E. Przekop

**Affiliations:** 1 Faculty of Materials Science and Engineering, Warsaw University of Technology, ul. Wołoska 141, 02-507 Warsaw, Poland; marta.dobrosielska@pw.edu.pl (M.D.); paulina.kozera@pw.edu.pl (P.K.); rafal.kozera@pw.edu.pl (R.K.); 2 Department of Non-Food Products Quality and Packaging Development, Institute of Quality Science, Poznań University of Economics and Business, al. Niepodległości 10, 61-875 Poznań, Poland; 3 Faculty of Chemistry, Adam Mickiewicz University in Poznań, 8 Uniwersytetu Poznańskiego, 61-614 Poznań, Poland; martakolodziejczak97@gmail.com (M.K.); julia.glowacka@amu.edu.pl (J.G.); 4 Centre for Advanced Technologies, Adam Mickiewicz University in Poznań, 10 Uniwersytetu Poznańskiego, 61-614 Poznań, Poland; ewa.gabriel@amu.edu.pl (E.G.); m.jalbrzykowski@pb.edu.pl (M.J.); 5 Faculty of Mechanical Engineering, Bialystok University of Technology, ul. Wiejska 45c, 15-351 Bialystok, Poland; krzysztof.kurzydlowski@pw.edu.pl

**Keywords:** polyamide 11, diatomite, mechanical properties, particle size, biocomposite

## Abstract

Amorphous diatomite was used as a filler for a thermoplastic polymer of polyamide 11 obtained from natural sources. The diatomite particles of different sizes were previously fractionated by sedimentation to obtain powders with varying particle size distribution, including powders with or without frustule particles, crushed, uncrushed or agglomerated. Biocomposites containing 2.5, 5, 10 and 20% filler were tested for their mechanical properties, including tensile strength, flexural strength and impact strength. In addition, a particle size analysis (by Dynamic Light Scattering, DLS) was performed and the dispersion of the filler in the polymer matrix (Scanning Electron Microscopy, SEM), thermal parameters (Differential Scanning Calorimetry, DSC, and Dynamic Mechanical Analysis, DMA) were determined. Testing showed that biocomposites modified with diatomaceous earth have a higher mechanical strength than the reference system, especially with larger amounts of the filler (10 and 20%), e.g., the tensile strength of pure PA11 is about 46 MPa, while 20OB and 20OF 47.5 and 47 MPa, respectively, while an increase in max. flexural strength and flexural modulus is also observed compared to pure PA11 by a maximum of 63 and 54%, respectively Diatomaceous earth can be obtained in various ways—it is commercially available or it is possible to breed diatoms in laboratory conditions, while the use of commercially available diatomite, which contains diatoms of different sizes, eliminates the possibility of controlling mechanical parameters by filling biocomposites with a filler with the desired particle size distribution, and diatom breeding is not possible on an industrial scale. Our proposed biocomposite based on fractionated diatomaceous earth using a sedimentation process addresses the current need to produce biocomposite materials from natural sources, and moreover, the nature of the process, due to its simplicity, can be successfully used on an industrial scale.

## 1. Introduction

**Figure 1:**
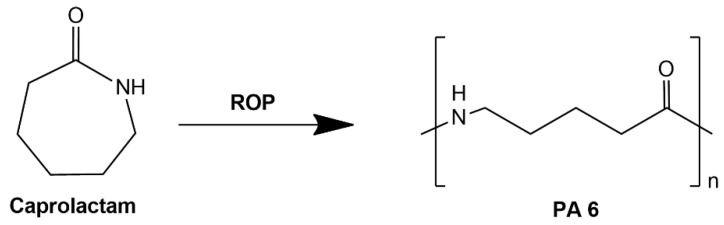
The reaction scheme for obtaining polyamide 6 from caprolactam.

Polyamide 11 (PA 11) is a thermoplastic polymer obtained from renewable sources derived from castor oil. Compared to polyamide 12, the production of PA 11 is by far more environmentally sustainable as it utilizes natural oil derivatives. In addition, PA 11 has excellent heat resistance, is stable under natural weather conditions and is characterized by high mechanical strength on the level comparable to that of PA 12 (Table 1). Since it is derived from natural sources, polyamide 11 is also biodegradable, which reduces its carbon footprint. Considering the abovementioned advantages, PA 11 is a good answer to traditional oil-derived polymers used, including the commonly used PA 12, in terms of environmental protection [1,2]. Polyamides are polymers whose chain structure contains amide groups (R-CO-NH-R’), widely used in many industries, such as automotive [3], aerospace [4], 3D printing [5], production of housings for power tools and machinery [6]. The polyamide varieties most widely used and discussed in the literature include: PA 6, PA 6,6, PA 12 and PA 11. The properties of the selected polyamide grades are compiled in Table 1. Polyamides can be obtained by polycondensation of dicarboxylic acids and diamines (PA 6,6), ω-aminocarboxylic acids (PA 11, PA 12) and by the ring opening reaction (ROP) of lactams (PA 6) [7]. Osváth, Zsófia, et al., in their review [8], described the recent developments in the production of PA 6 and composites thereof, including the thermoplastic resin transfer molding (TRTM) process, which is a promising method for large-scale fabrication of reinforced PA 6 composites. Polyamide 12 is produced on an industrial scale from lauric lactam by ROP. Due to the increasing environmental pollution and the depleting crude oil resources, the extraction of which could become economically unviable, alternative methods of polyamide synthesis are being sought worldwide. Evonik has developed a biotechnology for the production of PA 12 using ω-aminolauric acid derived from palm kernels [9]. Research is also being conducted on the synthesis of Bio-PA 6 as described by Oelmann and Meier [10]. The reaction scheme for the production of polyamide 6 is shown in Figure 1.

**Figure 2:**
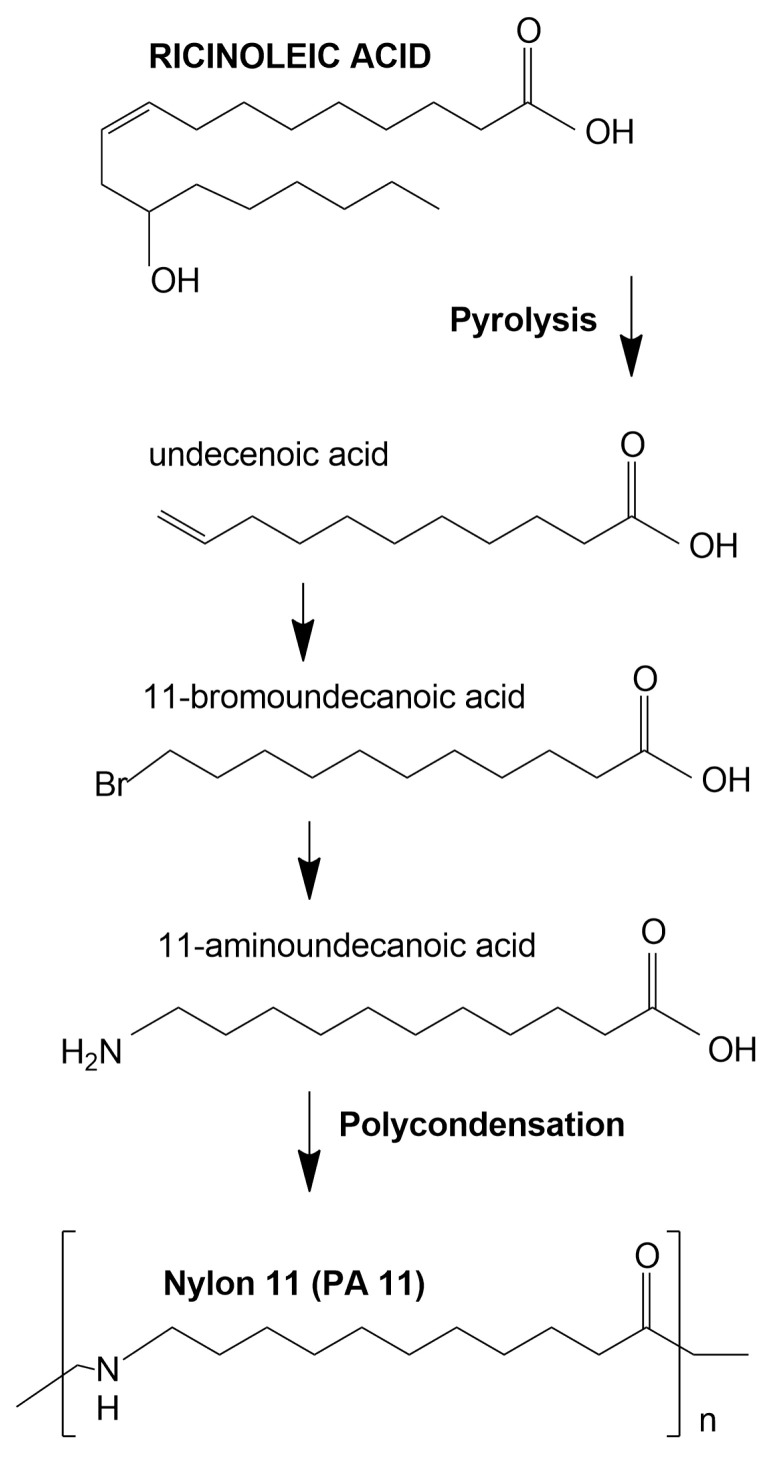
The reaction diagram for obtaining polyamide 11 from castor acid.

**Figure 3:**
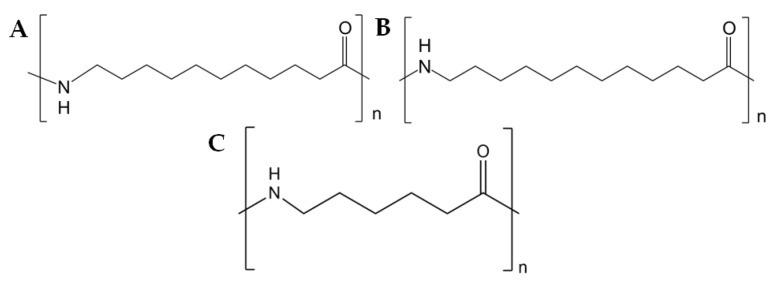
Monomers of polyamides; (**A**)—PA 11, (**B**)—PA 12, (**C**)—PA 6.

Nylon 11 [poly(imino-1-oxoundecamethylene)] has been produced on an industrial scale since 1940 by step-growth polymerization. The ricinoleic acid obtained from castor oil is successively pyrolyzed and brominated (HBr) in the presence of oxygen. The resulting 11-bromododecanoic acid is then treated with aqueous NH_3_, leading to the precipitation of a crystalline amino acid (11-aminoundecanoic acid). Polycondensation of this amino acid with aqueous extraction leads to obtaining polyamide 11 [11]. The utilization of castor oil for production of PA 11 renders it a bio-based polymer. The reaction scheme for the production of polyamide 11 is shown in Figure 2. Monomers of individual polyamides are presented in Figure 3.

Polyamide 11 can be processed in several ways. Among the most popular are injection molding and extrusion. In addition, rotational molding is a relatively new form of processing [18]. PA 11 is also successfully used in 3D printing as a powder for selective laser sintering (SLS) [19,20]. Due to its very good mechanical and chemical properties, it found applications for fabrication of components of both underwater and land-based pipelines, plastic car parts and housings for electronic equipment. It is also used as a coating material and in medicine [21,22].

As with other polymers of technical importance in structural applications, PA 11 has a long record on preparation of composites thereof containing various types of reinforcing phases. PA 11 reinforced with glass fibers (PA 11GF) is commercially available. Other examples cover the use of carbon fibers [23], basalt fibers [24] or graphene, the latter being introduced to increase wear resistance of coating-application-dedicated polyamide composites [25]. However, as mentioned above, due to its environmental friendliness and biodegradability, the most favorable modification procedures for PA 11 are carried out with natural raw materials. Polyamide can be reinforced with bamboo fibers [26], basalt/flax interwoven fibers [27], cellulose fibers [28], lignocellulosic pine fibers [29] or beech fibers [30]. Such biofillers are considered to be economically viable, less-contributing to the carbon footprint, and environmentally non-burdening when reaching material end-of-life [31]. The development of novel, biopolymer-based and/or biofiller containing, high performance biocomposites, constituting a large part of the bioplastics group, is an important trend in the development of circular economy [32,33].

Among the biofillers group, one deserving special attention is diatomaceous earth (DE), or in short, diatomite. It is a leftover mineral material constituting of frustules of dead diatoms, with particle size from single up to hundreds of micrometers. From a physicochemical point of view, it can be seen as porous microsilica doped with compounds of Al, Fe, Mg, Ca and traces of other elements [34]. Diatomite is considered biofriendly, non-toxic, and used for multiple applications, including as an adsorbent and filtering medium in wastewater treatment and other purification procedures, cement production and as a polymer filler [35]. In our previous works, we tested diatomaceous earth as a filler in different polymer systems, including epoxy composites [36,37], injection-molded PLA [38] and PLA-based composite filaments for FDM 3D printing [39].

In this work, we developed methodoloxy of preparation of PA 11/DE biocomposites and tested their processing behavior and service performance. For this purpose, polyamide 11 was filled with an amorphous diatomaceous earth which was previously fractionated by sedimentation, so that fractions with varied diatom grain sizes could be obtained. Biocomposites containing 2.5, 5, 10 and 20% of the filler (neat and fractionated diatomite) were prepared by extrusion and injection molding. The biocomposites were tested for mechanical strength, processing properties, heat resistance and filler dispersion in the polymer matrix. The tested samples were conditioned at room temperature, 23 °C (RT).

## 2. Materials and Methods

### 2.1. Materials

Polyamide 11 (Rilsan^®^ RE31840 Natural) (density = 1.03 g/cm^3^, MFI index = 30 g/10 min) in pellet form was purchased from Arkema (Colombes, France). Diatomaceous earth (Perma-Guard, Bountiful, UT, USA) in the form of powder was derived from diatomite deposits.

### 2.2. Preparation of Diatomite

**Figure 4:**
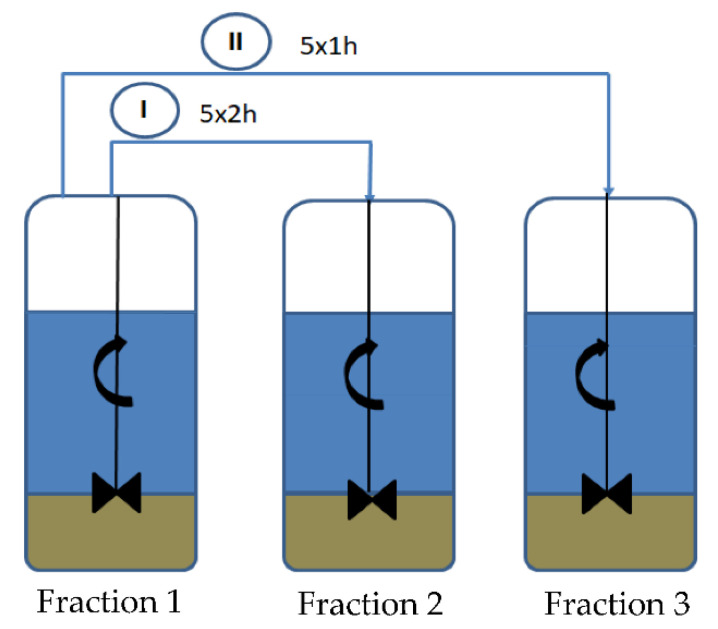
Fractionation of diatomite: I—5-fold sedimentation, 2 h each time; II—5-fold sedimentation, 1 h each time.

The original diatomaceous earth was fractionated in a similar way as in the previous works [36,37,38,39], according to Figure 4. Amorphous diatomite weighing 15 kg was placed in barrel 1, which was filled to a volume of 140 L with demineralized water. The whole mixture was mixed with a mechanical stirrer for 3 min and sedimented for 2 h. After this time, the supernatant liquid was pumped into the next barrel (2) with a pressure booster pump. The sludge remaining in the first barrel was again filled up with demineralized water and the washing was performed in the same way. The rinsing process was repeated 5 times, and the supernatant liquids (barrel 1) were pumped to obtain suspended solids in barrel 2. Another series of washes of the remaining sludge in the first barrel was carried out. This time the procedure included 1 h of sedimentation. Barrel number 1 with the sediment was filled up with demineralized water to a volume of 140 L and stirred for 3 min with a mechanical stirrer. The suspension was allowed to settle for 1 h and then the supernatant liquid was pumped into barrel 3. The procedure was repeated 5 times and a suspension in barrel 3 was obtained from the liquids poured together. One week after the end of fractionation, the remaining supernatant liquids were pumped out of barrels 2 and 3. The sludge from barrels 1, 2 and 3 were dried at 70 °C for 24 h. Eventually, 3 fractions of diatomite with different particle size distributions were obtained. Table 2 shows the weights of the obtained sludges after the respective number of washes and their percentage in relation to the used diatomite (15 kg). The loss of material was due to the removal of supernatant liquid, which partly contained dispersed diatomite particles. Fraction 2 of the washed diatomaceous earth was used to obtain the polyamide-based biocomposites, because a large amount of diatomite was obtained and the particle size distribution is significantly different from the original diatomite, which also allowed us to investigate the effect of particle size on the properties of the biocomposites produced. 

### 2.3. Extrusion of Biocomposite

The extrusion of biocomposites was carried out on a process line RHEO DRIVE 16 from Haake PolyLab OS (Thermo Scientific) (Waltham, MA, USA). The process was carried out with a twin-screw extruder. Before extrusion polyamide 11 was dried out for 8 h in 90 °C. A mixture of PA 11 and original diatomaceous earth was fed into the hopper in portions (of 50 g) at four concentrations of 2.5%, 5%, 10% and 20%, respectively. The screws were used to plasticize and stir the materials. The extrudates were dried at 70 °C for 24 h, and then ground in WANNER C17.26 sv plastic mill (Łódź, Poland). Four compositions of polyamide 11 with diatomaceous earth were obtained in concentrations of 2.5%, 5%, 10% and 20%. The remaining biocomposites were extruded in a similar way. The parameters of the extrusion process are shown in Table 3.

### 2.4. Injection Molding—To Obtain Standardized Measurement Paddles

**Figure 5:**
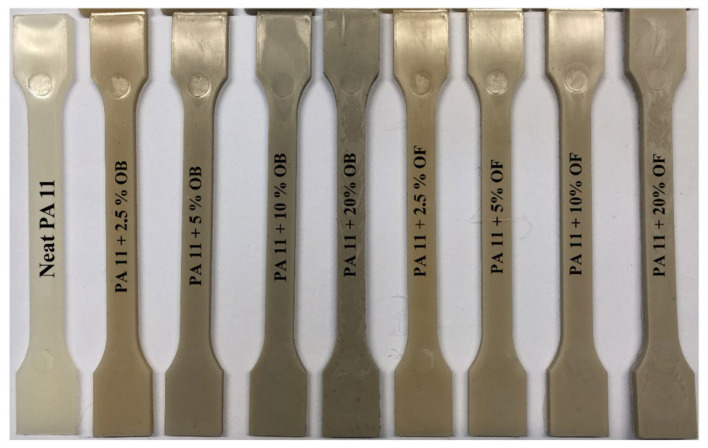
Biocomposites obtained by injection.

The injection was performed on a column-free e-victory 170/80 injection molding press from Engel (Warsaw, Poland). Table 4 shows the process parameters. A holding pressure of linear increment over time was applied. The mold temperature was maintained at 80 °C. Standard measuring preforms of the type 1A were obtained according to ISO 527 (Figure 5). Finally, 4 biocomposite types and a reference system (Table 5) were obtained.

### 2.5. Characterization Methods

Paddle-shaped specimens prepared in accordance with EN ISO 527-1 were used for the strength tests: 2012 and EN ISO 178:2010. Testing was carried out on the INSTRON 5969 strength testing machine with a peak load force of 50 kN (Instron, Norwood, MA, USA). The traverse rate for the tensile strength measurements and flexural strength measurements was set at 5 mm/min. Charpy impact strength (with V-notch) was performed using Instron’s Ceast 9050 impact device with pendulum hammer with a maximum energy of 25 J according to ISO 179-1:2000 (Instron, Norwood, MA, USA). The impact strength test was conducted in accordance with PN-EN ISO 178-1. The notch was machined and positioned in the center of the preform according to ISO 179-1/1eA. A V-shaped 2 mm-deep notch with a 45° angle was produced. According to the standard, the impact direction was aligned with the edge. Dynamic mechanical analysis (DMA) was performed using a Q800 DMA (TA Instruments, USA) in dual cantilever mode according to ASTM D4065-01. Three rectangular specimens with 60 mm length and 10 mm width were cut from each biocomposite and used in the test. The analysis was conducted from 0 to 130 °C with a heating rate of 3 °C/min at a frequency of 1 Hz and an amplitude of 30 µm. The glass transition temperature (Tg) was determined from the peak value in the storage modulus using TA Universal Analysis software. The powder morphology was characterized using a scanning electron microscope (SEM, TM 1000 Hitachi, Japan) operating at an applied voltage of 5 kV. Before observations with the SEM, the surfaces of the specimens were sputtered with a gold-palladium layer for 90 s at a current of 10 mA and voltage of 2 kV. The melt flow rate (MFR) was measured using the Instron CEAST MF20 melt flow tester (Instron, Norwood, MA, UDA), which complies with EN ISO 1133 at 235 °C for a load of 2.16 kg. The size of the diatoms used to prepare the biocomposites was measured with Mastersizer 3000 (Malvern Instruments Ltd., Malvern, GB). The measurements were made for the samples in water suspension (Hydro EV attachment). The parameters of the measurements for the wet samples: stirrer speed: 2330 RPM; ultrasound power: 70%. Thermal properties of materials were studied using a Q1000 Differential Scanning Calorimeter (TA Instruments, New Castle, DE, USA). Samples with a weight of 8.0 + 0.2 mg were placed in an aluminum hermetic pan. Firstly, the samples were equilibrated at −90 °C, then heated to 230 °C with a scan rate of 10 °C/min, and cooled to −90 °C with a scan rate of 10 °C/min. Finally, they were heated again to 230 °C with a scan rate of 10 °C/min. The process was conducted in a nitrogen atmosphere (20 mL/min). Using the Universal V4.5A TA software, the glass transition temperature (Tg) was determined as the midpoint of the glass transition temperature range. The melting temperature was determined as the peak temperatures of cold crystallization and melting, respectively. Moreover, the degree of crystallinity was calculated. The heat of fusion of 100% crystalline Nylon 11 was equal to 206 J g^−1^ [11]. The viscosity of the biocomposite was determined by the capillary method and using the Instron CEAST SR10 Smart RHEO 1000 capillary rheometer. The measurement was carried out at 235 °C using a capillary tube with a diameter of 1 mm and a length of 20 mm. Viscosity was determined for 6 shear rates, that is, 10, 30, 100, 500 and 1000 (1/s). Images of the surface and fractures of the biocomposites were taken using KEYENCE VHX-7000 digital microscope (KEYENCE INTERNATIONAL, BELGIUM, NV/SA) with a VH-Z100R wide-angle zoom lens at 100× magnification. Images were taken with depth composition and 3D imaging. Total coaxial illumination was used. Mechanical properties, i.e., tensile breaking strength, flexural strength, impact strength and wetting angle were tested for biocomposites, which were previously conditioned at room temperature. Viscosity testing, fracture imaging (SEM) and thermal tests (DSC, DMTA) were performed on selected biocomposites, i.e., those containing 5 and 20% filler in order to compare the effects of low and high filling on rheological, mechanical and thermal properties.

## 3. Results

### 3.1. Particle Size Distribution of the Modifier (Determined Using DLS)

**Figure 6:**
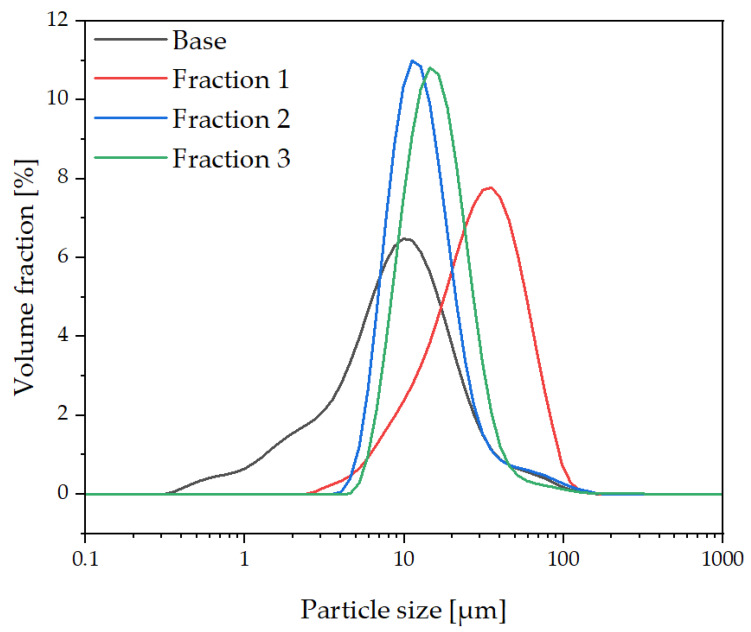
Particle size distribution of fractionated diatomite.

The fractionation of raw diatomaceous earth resulted in three fractions that differed in particle size distribution, as shown in Figure 6. The original diatomite has a particle size distribution ranging from 1 μm to about 100 μm with particles with an average size of approx. 10 μm making up the largest volume fraction. Fraction 1, which was washed 10 times, has particles ranging in sizes of approx. 3 to 100 μm, with the largest number of particles of about 40–50 μm, indicating that this number of washes resulted in the removal of low-diameter particles but enriched the material with large-diameter particles. Fractions 2 and 3 do not show any significant differences from each other. The two fractions have a significant proportion of particles ranging in size from 4 to about 35 μm and the elimination of ultrafine particles that remained suspended in the supernatant after washing the diatomite. This method of fractionating diatomite made it possible to obtain sediments with completely different particle size distributions. One of the resulting fractions had larger particles that were probably agglomerated, and the other contained no agglomerates and particles smaller than 4 μm, i.e., diatoms with crushed frustules. Similar results, i.e., partial separation of the crushed particles from the uncrushed and agglomerated particles, were obtained in previous work [39], which shows the repetition of the sedimentation, which is a process of deposition.

### 3.2. Rheology—Melt Flow Rate (MFR) and Capillary Rheology

**Figure 7:**
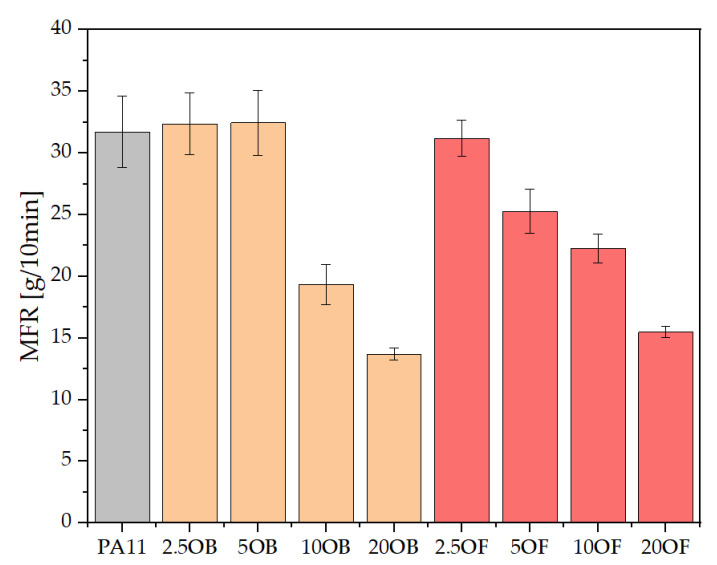
Melt flow rate (MFR) of biocomposites.

Melt flow rate testing showed that pure polyamide 11 has a relatively high MFR (about 30 g/10 min), which is consistent with literature reports [40]. The modification of PA11 with original diatomite at a low concentration, that is up to 5 wt%, does not cause significant changes in processing properties, while higher addition of modifier (10 and 20%) results in almost twice as low values (~20 and ~15 g/10 min, respectively) (Figure 7). The modification with fractionated diatomite and the addition of as little as 5% modifier resulted in a linear decrease in the MFR when the concentration was lowered, but the final values are similar to those for the original diatomite. The addition of up to 20% modifier does not cause any general difficulties in the processing of biocomposites, as the melt flow rate of 15 g/10 min is still sufficient for both extrusion and injection of small components, the standardized preforms studied in this work.

**Figure 8:**
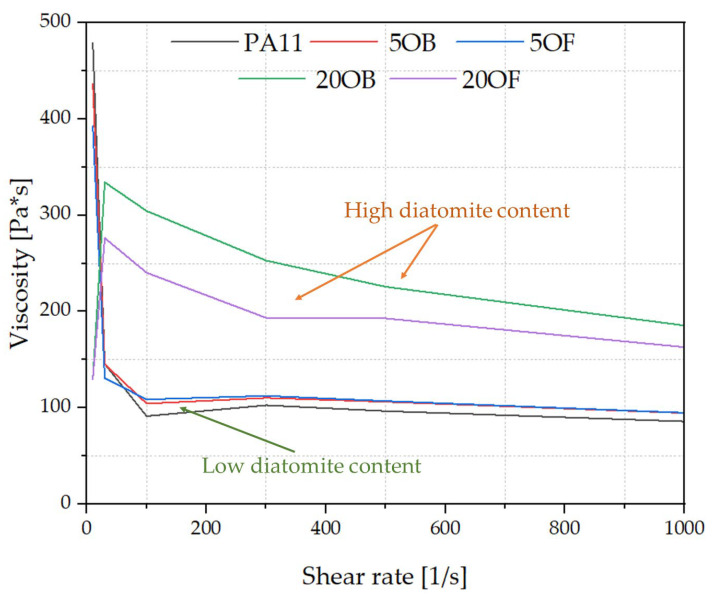
The viscosity of biocomposites of PA11 and diatoms after the injection (capillary rheology).

As expected, the reference polyamide 11 has the lowest viscosity of all tested samples (Figure 8). Filling the biocomposite to a concentration as low as 5% causes an increase in viscosity for the samples with both the original and the fractionated diatomite, although the effects are small at high shear rates. In addition, for low-filled systems, the viscosity is almost constant at a shear rate of 100–1000 1/s. Highly filled systems with 20 wt% filler exhibit a completely different relationship. They have a much higher viscosity than the others, on average more than double, with the system containing the original diatomite having a higher viscosity due to the proportion of micro and sub-micro fractions. A similar effect was described in a previous paper [36]. Diatom particles, due to their size, penetrate between the biopolymer chains and consequently disrupt their ordering. This effect results in smaller interactions between the chains, which reduce the viscosity of the biocomposites. In addition, the viscosity curves show a different tendency than low-filled systems. The shear thinning effect is more pronounced [41]. From the above correlations, a small addition of diatomite filler does not significantly worsen the viscosity of the biocomposite systems in the melt, while at high filler levels (20%) the use of sedimented filler can keep the melt viscosity at a lower level. Filler particle size also affects the viscosity of biocomposites. For 20% filler concentration, particle size control improved processability by reducing external friction. The addition of diatomaceous earth with a smaller particle size range (Fraction 2, Figure 6) resulted in lower viscosity compared to unfractionated diatomite.

### 3.3. Mechanical Tests

#### 3.3.1. Tensile Strength

**Figure 9:**
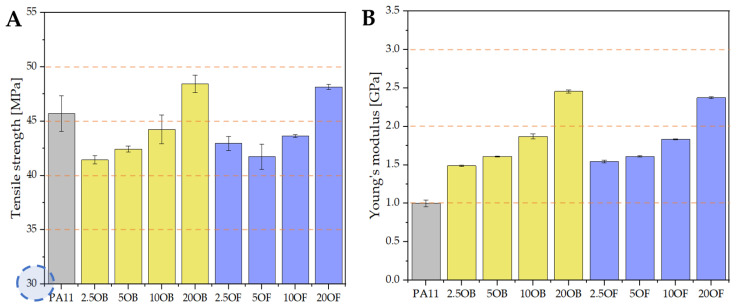
The results of the static tensile test are as follows: (**A**) the tensile strength and (**B**) the Young’s modulus of the biocomposites conditioned at room temperature (RT).

**Figure 10:**
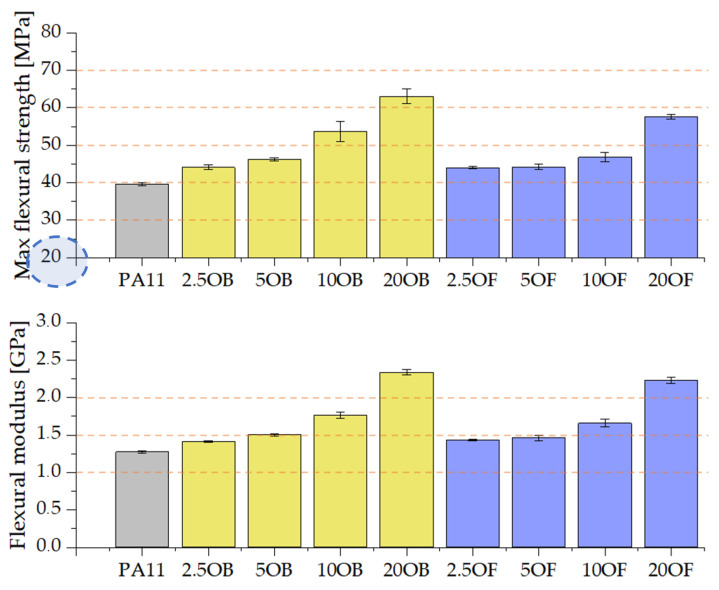
The results of static flexural strength test: maximum bending stress and modulus of elasticity.

**Figure 11:**
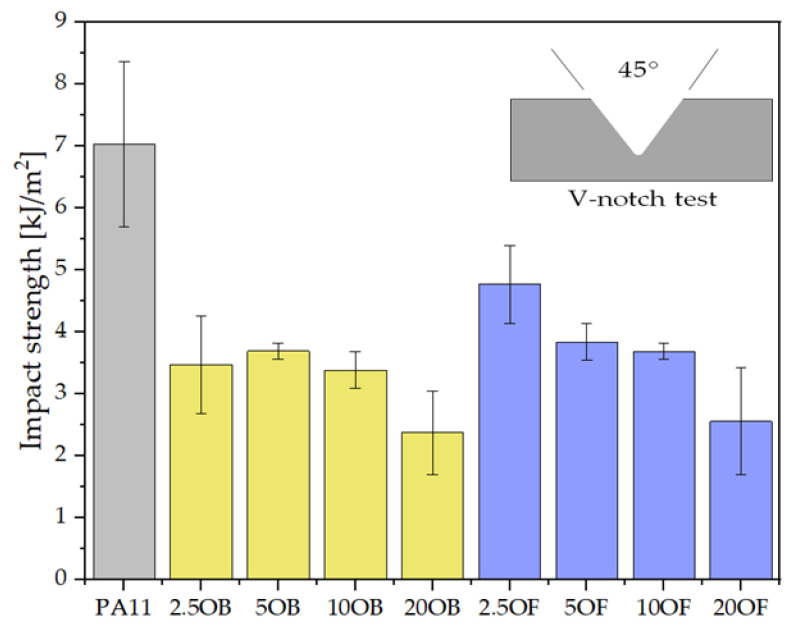
The results of the Charpy impact test (Charpy V-notch test).

Mechanical tests were carried out on biocomposites conditioned at room temperature, as shown in Figure 9, Figure 10 and Figure 11. As expected, modification of pure polyamide 11 with both original and fractionated diatomite resulted in a slight decrease in tensile strength for low filler concentration compared to neat PA11 (Figure 9). Both the tensile strength and the Young’s modulus increased with increasing filler concentration, with fractionation of diatomite resulting in slightly higher tensile strength values as soon as for the lowest concentration, compared to the original diatomite. By filling at a level of 20%, values were achieved that were higher than those of pure polyamide. This could indicate that it is important to eliminate the matrix effect on the mechanical strength of the polyamide in favor of the natural filler. In our previous work, the reinforcing effect of diatomite filler on polylactide [38,39] and epoxy resin [36,37] was also observed for different ways of obtaining composites. Due to the structure of diatoms, there can be an effect of penetration of biopolymer molecules into the filler particles; thus, strengthening the structure of the biocomposite. Diatoms acts asan excellent anchor where hydrocarbon chains can bond together strengthening the structure. There is also a limiting concentration of diatomaceous earth at which the structure strengthening effect begins to prevail, as observed in the form of increased tensile strength. For both base and fractionated diatomaceous earth, this concentration is more than 10% filler.

#### 3.3.2. Flexural Strength

The modification of the pure polyamide 11 with diatomaceous earth improved the mechanical parameters, including the flexural strength (Figure 10). Pure PA11 withstands a peak bending stress of 40 MPa, and has a modulus of elasticity of 1.25 GPa, which is the lowest measured value for the tested samples previously conditioned at room temperature. As the modifier concentration in the series increases, both parameters increase linearly. Although the particle sizes were different in the original diatomite compared to the fractionated diatomite, no significant differences were found in the flexural strength values for the corresponding samples in the series.

#### 3.3.3. The Charpy Impact Test (V-Notch Test)

The impact strength of pure polyamide 11 is relatively high, approximately 7 kJ/m^2^ on average. Filling the biocomposite with diatomite reduces the impact strength due to the nature of the filler (powder filler). The higher the filling ratio of the biocomposite, the lower the impact strength values, which is due to the fact that air is trapped in the diatom particles, causing early crack propagation (Figure 11). The more filler added, the greater the amount of air present.

### 3.4. Morphology of Biocomposite Surfaces

**Figure 12:**
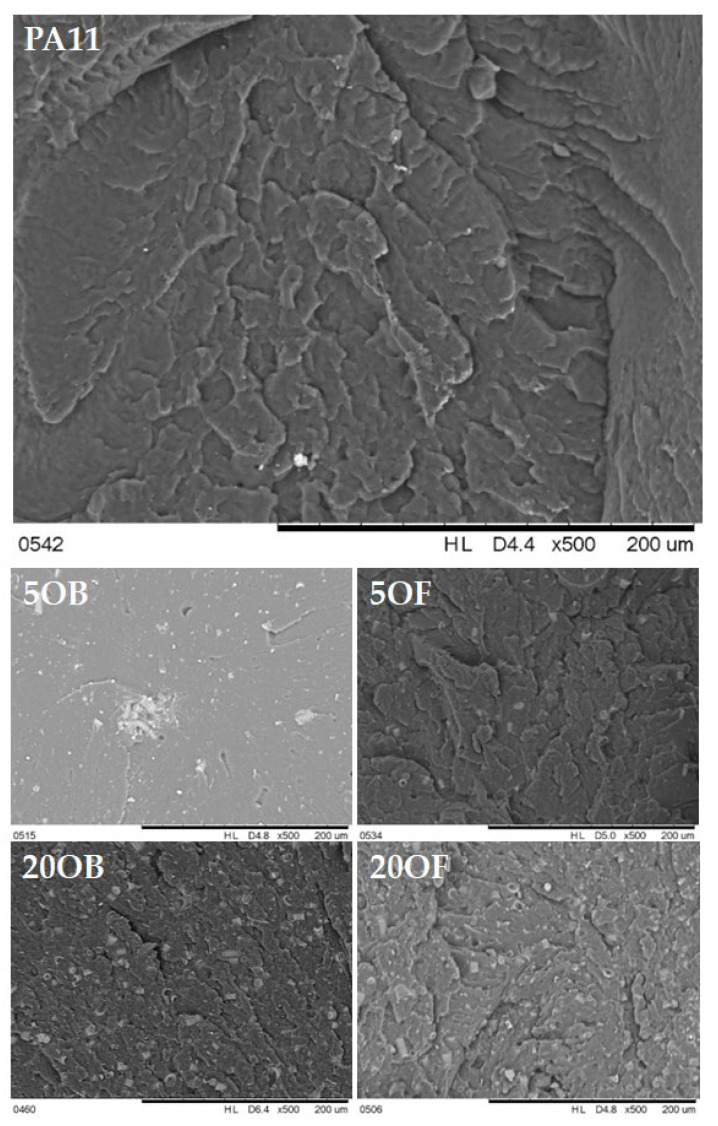
SEM images of biocomposite fractures.

Images of biocomposite fractures taken with a scanning electron microscope at a magnification of 200 μm are shown in Figure 12. Once fractured, the pure polyamide 11 delaminates and its structure remains homogeneous. Biocomposites containing diatomaceous earth show similar behavior. In most cases, numerous furrows are present on the fracture surfaces. Only the biocomposites modified with 5% original diatomite show a smooth fracture structure, without furrows. The diatomaceous earth is uniformly dispersed in the polymer matrix and no tendency to agglomeration is observed. SEM images confirm that the amount of diatom particles increases with the increasing concentration of the modifier.

**Figure 13:**
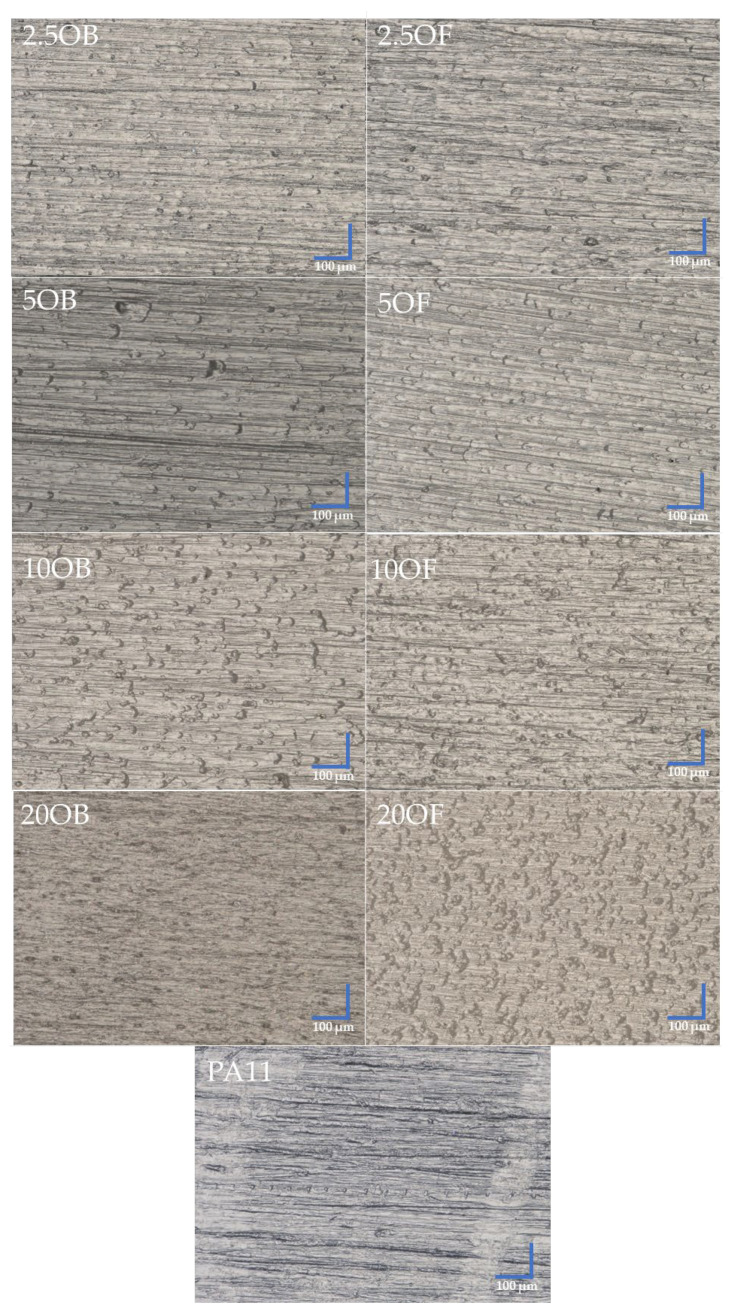
Surface of the biocomposites.

**Figure 14:**
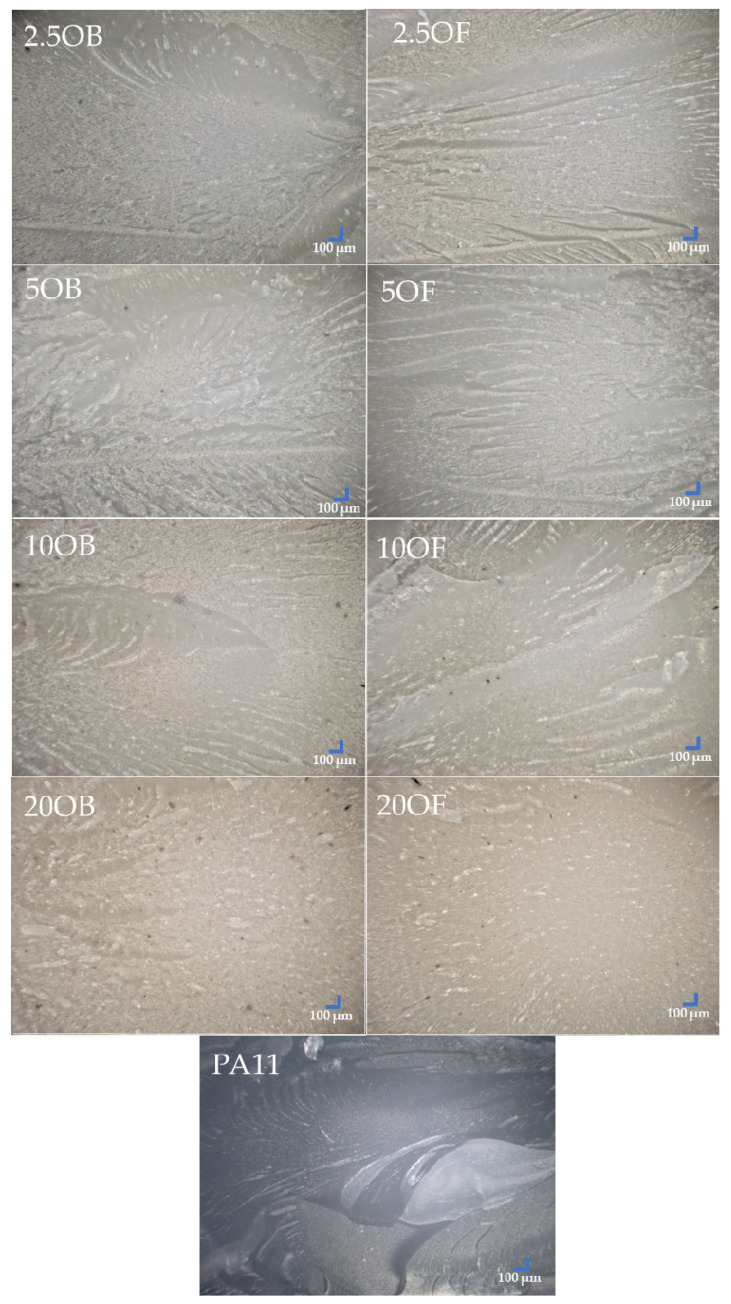
Biocomposite fractures after notched impact strength testing.

An optical microscope was used to image the surface of the biocomposites (Figure 13), and their fractures that occurred after the notched impact strength testing (Figure 14). The reference system used was pure polyamide 11 (PA11). Polyamide has a relatively smooth surface, but small furrows are locally present, indicating the relatively poor scratch resistance of the polyamide, which is mostly absent in the biocomposites filled with diatomite. The samples modified with original diatomite show color differences both between concentrations within the system and compared to the samples filled with fractionated diatoms, possibly due to the different particle arrangement (agglomeration) of the original diatomite, which does not occur for fractionated diatomite due to the narrower range of particle sizes. As the concentration of the original or fractionated filler increases, the surface of the biocomposite becomes irregular and numerous protrusions are formed. This effect is more evident when only 10% of the original diatomite is added, while irregularities appear on the surface when the filler concentration is only 20%. Examination of the biocomposite fractures showed that the fractionation of diatomite had no significant effect on the type or brittleness of a crack. Regardless of the modifier used, the fractures are very similar. At concentrations between 2.5% and 10%, furrows and numerous “wrinkles” formed in the material when the biocomposite particles were pulled out. For highly filled samples (20%), the effect is much less. There are a few changes in the material structure that can be attributed to high-energy impact.

**Figure 15:**
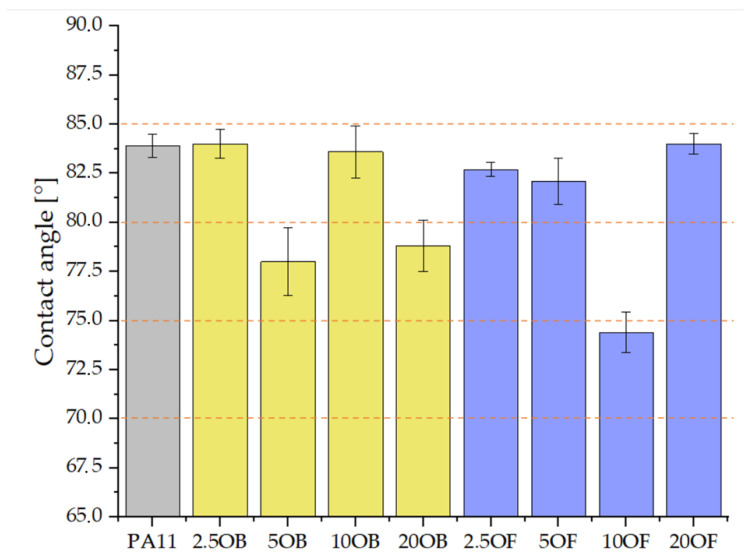
Contact angles of biocomposites’ surfaces.

Gloss testing was performed in a 60° geometry (Table 6). Pure polyamide 11 exhibited surface gloss values of 31.5 GU, making it a medium gloss system. The addition of diatomaceous earth resulted in a decrease in gloss and the formation of a surface towards matte. The gloss of the biocomposites decreased linearly as the filler concentration of the base diatomaceous earth increased. This was different for diatomaceous earth fractionation, where the highest results of 35.7 and 26.1 GU were recorded for biocomposites filled at 5 and 10%, respectively. The surface of all tested samples, both pure polyamide 11 and biocomposites modified with diatomaceous earth, have hydrophilic character due to the value of wetting angle below 90° (Figure 15). No linear relationship between filler content and surface character was observed. Both fractionated and base diatomaceous earth show similar values of 75–84°. High filling of polyamide 11 with diatomaceous earth (20%) did not result in loss of surface properties, especially for fractionated DE.

### 3.5. Thermal Tests

**Figure 16:**
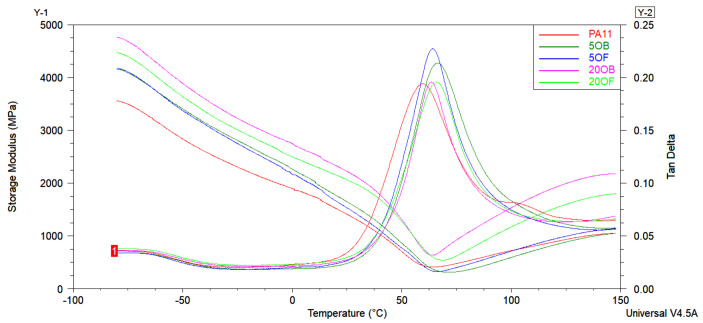
The results of the storage modulus and tan δ of PA11/diatoms biocomposites.

The DMA thermograms of the PA11 biocomposite materials were created to determine the stiffness of the materials with the change in the temperature. As shown in Figure 16, the evolution of the storage modulus (E’) and tan δ of PA11 and the biocomposites as a function of temperature showed a transition in the studied range, related to the glass temperature of the polymer matrix. The measured values of the Tg are presented in Table 7.

Figure 16 shows how the addition of different contents and different types of diatomaceous earth influenced the storage modulus (E’) of biocomposites based on PA11 and diatoms. For comparison, the results were compared with the Tg of pure PA11. No considerable differences were observed in the Tg of the biocomposites by the effect of diatom content. Only a distinct increase in the Tg value was observed. However, the addition of 5 weight fractions of OB and OF to biocomposites caused a higher change in the glass transition temperature than the application of 20 wt% components. The fractionated diatomaceous earth increases the Tg value by 1.3° compared to the original when 20 wt% diatomite was added. Thus, the fractionation of diatoms has a beneficial effect on the thermal properties of PA11-based biocomposites, especially in the higher concentration range. The addition of diatoms into the PA11 polymer allowed for obtaining stiffer material with higher values of storage moduli due to the stiffening effect of the reinforcement. As it was found in [42], an increase in the weight fraction of the reinforcement causes an increase in the storage modulus of the biocomposite. Once the temperature was over the Tg, the storage modulus values were decreased, indicating high mobility of the polymer molecules corresponding to the amorphous phase of the PA11. The presence of diatoms in the biocomposite structure was found to significantly reduce the mobility of the polymer chains at temperatures higher than its Tg.

**Figure 17:**
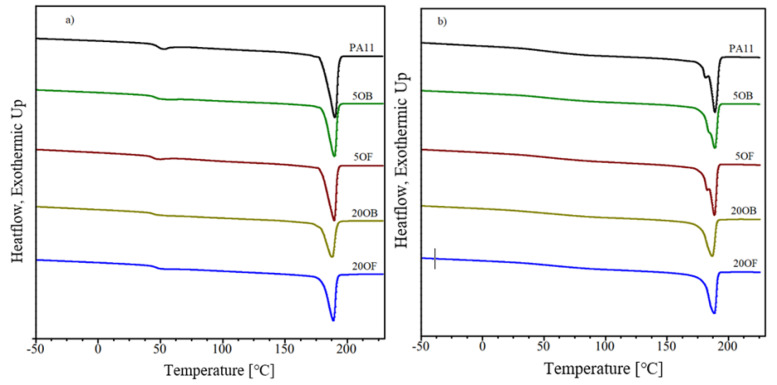
DSC curves of unmodified PA11 and biocomposites based on PA11 with different weight fractions of diatoms obtained during first heating procedure (**a**) and second heating procedure (**b**), respectively.

DSC analysis was performed to investigate the thermal behavior of PA11 and PA11/diatom biocomposites. Figure 17 shows the DSC thermograms of PA11 and PA11/diatom biocomposites during the first heating and the second heating stage, respectively, and Table 8 shows the measured values. With increasing temperature, DSC curves of the first heating showed two thermal characteristics: a glass transition temperature (Tg) of nearly 50 °C, and an endothermic melting pI(Tm) of nearly 190 °C. It was found that the addition of diatoms resulted in a slight decrease in Tg and Tm temperatures. The inverse dependency was obtained during cooling. The use of diatomaceous earth had an influence on the increase of the crystallization temperature. For the second heating curves, it was observed that the unmodified PA11 and 5B and 5OF biocomposite showed two distinct melting peaks (Tm1 and Tm2) while 20OB and 20OF biocomposite had a single melting peak. It was found that PA11 and 5B and 5OF biocomposite presented the main peak at about 188 °C, preceded by a shoulder with a maximum at about 182 °C as rearrangement in the structure. Depending on temperature and processing conditions, PA11 has different crystalline structures which can be interconverted. This double peak corresponded to the melt-crystallization process of the γ phase to the α’ crystalline form of PA11. The secondary peak seemed to decrease when the diatomaceous earth content was increased in the biocomposite material [34,35,36,37,38,39,40,41,42,43,44,45]. In addition, the peak of the glass transition temperature broadened considerably, and it is difficult to determine the Tg from the DSC curves during the second heating. The heat-of-fusion values determined from the second heating stage were used to obtain the crystallinity values, and these are tabulated in Table 8. Although the changes were very small, the crystallinity content consistently appears to increase with the content of diatoms. Moreover, higher degrees of crystallinity were obtained for composites with fractioned diatoms. Similar results were obtained in [46], which also observed an increase in the degree of crystallinity depending on the rise in the content of the additive to the polymer matrix.

## 4. Conclusions

Fractionation of the original diatomite using a simple vessel system and the sedimentation phenomenon allowed the obtaining of fractions with different particle size distributions. The resulting fractions differ in their content of crushed, uncrushed, and agglomerated diatom particles. The use of a sedimented and submicron-free fraction resulted in a lower increase in the viscosity of the melt, which is an advantageous effect from a processing point of view. Both the original diatomaceous earth and fraction 2, which were used as biocomposite fillers, do not tend to agglomerate and thus do not worsen the mechanical parameters. A suitable dispersion of the filler in the polymer matrix eliminates the problem of too early crack propagation. The effect is significantly less prominent in the case of tensile strength, though, as a considerable drop in its values is observed for 2.5–10% loadings, and only at 20% loading the biocomposites are characterized by statistically improved tensile strength. As the filler concentration increases, the above parameters improve significantly. The modification of polyamide 11 with diatomite also led to an increase in the thermal parameters of the biocomposites. An increase in Tg of 5.8 °C was observed. A lower filler concentration has a more favorable effect on the thermomechanical stability of the biocomposites. High filling of diatomaceous earth (20%) did not result in a decrease in the hydrophilic-hydrophobic character of the surface, especially for fractionated DE.

## Figures and Tables

**Table 1:** Comparison of the properties among PA 11 [12,13,14], PA 12 [14,15,16] and PA 6 [14,17].

	PA 11	PA 12	PA 6
Tc (°C)	142–158.8	147–148	191.8–193.2
Tm (°C)	176–198	174–185	220–260
Tg (°C)	35–46	55	50–75
Polydispersity Mm/Mn	1.72–2.50	1.54–3.5	1.7–2.4
Crystallinity (%)	16.8–36	30–52	24.4–50
Tensile strength (MPa)	40.53–42	50–70	74–106
Tensile modulus (MPa)	1288.83–1300	1.400–1.600	780–3.000
Izod impact strength (kJ/m^2^)	13.49	30	17
Charpy impact strength (kJ/m^2^)	5–15	6–25	3.5–82
Flexural strength (MPa)	~45	60.7	100
Flexural modulus (MPa)	290–1060	360–1260	2.600
Shore D hardness	64–75	61–79	78.5–82

**Table 2:** The weight of sediments obtained.

	Weight of Sediment (kg)	Percentage (%)	Times Washed
Fraction 1	1.785	11.90	10
**Fraction 2**	**10.561**	**70.40**	**5**
Fraction 3	0.952	6.35	5
Total	13.298	88.65	-

**Table 3:** The parameters of the extrusion process.

**Temperature at heating zones (°C)**	**Nozzle**	**TS6**	**TS5**	**TS4**	**TS3**	**TS2**	**TS1**
	235	250	250	255	255	250	250
**Torque, M (Nm)**		**Nozzle pressure, P_50_ (bar)**			**Revolutions per minute, N (1/min)**		
20–78		0.6			14		

**Table 4:** Injection process parameters.

**Temperature (°C)**	**Nozzle**	**Zone 1**	**Zone 2**	**Zone 3**	**Traverse**
	215	220	215	210	40
**Mold closing force (kN)**	**Clamping time (s)**		**Cooling down time (s)**	**Screw diameter (mm)**	
800	4		30	25	

**Table 5:** Biocomposites to be tested.

Biocomposite Type	Filler Concentration	Abbreviation
PA11 + original diatoms	2.5%	2.5OB
	5%	5OB
	10%	10OB
	20%	20OB
PA11 + fractionated diatoms	2.5%	2.5OF
	5%	5OF
	10%	10OF
	20%	20OF
PA11	-	PA11

**Table 6:** Gloss of biocomposite surfaces.

	Gloss 60° (GU)	SD (GU)		Gloss 60° (GU)	SD (GU)
2.5OB	24.53	0.50	2.5OF	23.00	0.08
5OB	21.90	0.54	5OF	35.70	1.13
10OB	17.50	2.12	10OF	26.10	0.75
20OB	15.07	0.74	20OF	16.57	0.12
PA11		31.50		1.93	

**Table 7:** Summary of the determined values of the glass transition temperature from storage modulus E’ curves.

Sample	T_g_ (◦C)
PA11	50.0
5OB	55.8
5OF	55.7
20OB	52.7
20OF	53.9

**Table 8:** Thermal characteristics of PA11 and biocomposites based on PA11 with different weight fractions of diatoms.

	1st Heating			Cooling		2nd Heating			
Sample	T_g_ (°C)	T_m_ (°C)	ΔH_m_ (J/g)	T_c_ (°C)	ΔH_c_ (J/g)	T_m1_ (°C)	T_m2_ (°C)	ΔH_m_ (J/g)	X_c_ (%)
**PA11**	48.7	190.0	56.9	163.9	53.2	181.4	189.0	51.6	25.0
**5OB**	46.8	189.5	44.6	167.6	50.4	182.6	189.0	45.0	22.9
**5OF**	44.0	189.5	48.7	167.1	49.11	182.8	188.6	50.3	25.7
**200B**	44.6	187.6	36.4	167.8	50.7	-	186.6	44.1	26.7
**20OF**	46.6	188.7	39.2	169.7	51.5	-	188.4	44.7	27.1

## Data Availability

The data presented in this study are available on request from the corresponding author.
